# Progressive Familial Intrahepatic Cholestasis: A Rare Cause of Cirrhosis in Young Adult Patients

**DOI:** 10.1155/2015/428638

**Published:** 2015-06-02

**Authors:** Gavin R. Sun, Michele Burns

**Affiliations:** ^1^Department of Internal Medicine, University of Calgary, Calgary, AB, Canada T2N 4N1; ^2^University of Calgary, Rockyview General Hospital, Calgary, AB, Canada T2V 1P9

## Abstract

Hepatic cirrhosis is an important cause of morbidity and mortality. An unusual case of cirrhosis and portal hypertension in an 18-year-old patient secondary to Progressive Intrahepatic Cholestasis is discussed. The clinical and biochemical findings are discussed and a clinical approach to determining the underlying etiology of cirrhosis is outlined. Significant complications of portal hypertension include ascites, spontaneous bacterial peritonitis, hepatorenal syndrome, varices, and hepatic encephalopathy. A clinical approach to these complications of cirrhosis is presented. Progressive Familial Intrahepatic Cholestasis (PFIC) is a rare congenital metabolic abnormality. There are 3 subtypes and Type 3 PFIC commonly presents in late adolescence and early adulthood. Clinical and laboratory findings as well as management for the condition are described.

## 1. Introduction

An 18-year-old male was admitted to the Medical Teaching Unit (MTU) after presenting to the Emergency Department with an 8-day history of feeling lethargic and generally unwell. He reported worsening nausea but denied experiencing any fevers, chills, cough, muscle aches, night sweats, or vomiting. He was admitted due to one episode of tarry stools the day before admission and revealed that he had recently taken Ibuprofen for 2-3 days for a strained thigh muscle. He denied any symptoms of acid reflux.

## 2. Background

The patient recalled recurrent epistaxis for the preceding 2-3 years and had experienced a prolonged episode of 1-2 hours in the week prior to presentation to the ER. He denied any abdominal pain but did note increasing distention. He had been seen in consultation by Pediatric Gastroenterology 2 years earlier for persistent watery diarrhea. No melena or hematochezia was noted at that time. Hemoglobin levels were within normal range and no abnormalities were seen on gastroscopy or colonoscopy. There also appeared to be a history of cognitive delay. The patient's father reported that his son only learned to speak at age of 6 and, though never formally assessed, has had difficulties with memory, expression, and comprehension.

The patient was not taking any medications and had no known allergies. His father had been diagnosed with thalassemia trait. There was no family history of lymphoma, thrombophilia, leukemia, gastric cancer, or colon cancer. There was no history of smoking, alcohol consumption, or use of recreational drugs. There was no recent travel. He was employed as an automechanic apprentice.

On physical examination, the patient appeared pale with pale conjunctivae. His temperature was 37.4°C, pulse 97 beats/minute, and respiratory rate 18 per minute. His blood pressure was 111/59 and oxygen saturation was 98% on room air. Cardiovascular exam revealed a jugular venous pressure 5 cm above the sternal angle. S1 and S2 were normal but a 2/6 systolic murmur was audible over the precordium without distal radiation. Chest examination was remarkable for decreased breath sounds on the right side but absence of adventitious sounds. His abdomen was generally distended and was tender in the right upper quadrant. The liver edge was palpable and upon percussion of most inferior interspace on the left anterior axillary line there was a shift from tympany to dullness, indicating a positive Castell's sign. Shifting dullness was noted. Palpable lymph nodes were not noted. Central nervous exam was grossly normal [1].

## 3. Investigations

Significant initial laboratory findings revealed a severe microcytic anemia with elevated liver enzymes and evidence of liver dysfunction (hemoglobin 43 × 10^9^ g/L, ALP 589 U/L ALT 173 U/L, GGT 148 U/L, albumin 47 g/L, LD 132 IU/L, and total bilirubin 47 *μ*mol/L). Serum electrolytes, calcium, magnesium, phosphate, urea and creatinine, TSH, and CRP were in normal range. Serum lipase was 31 U/L. The hemoglobinopathy screen was negative. Iron studies confirmed iron deficiency (3 *μ*mol/L, TIBC 90 *μ*mol/L, transferrin saturation 0.03%, and ferritin 5 *μ*g/L). Serum protein electrophoresis was normal with no dominant protein bands. Alpha-1 antitrypsin level was 2.17 *μ*mol/L.  Table 1 summarizes the infectious work-up.

Diagnostic imaging included a CT of the chest, abdomen, and pelvis. Hepatosplenomegaly was reported and the splenic span was 22 cm. A nodular liver was visualized but no distinct masses were seen. Bilateral pleural effusions and marked abdominal ascites were also noted. As a result of the patient's iron deficiency anemia, he was transfused 3 units of packed red blood cells on the day of admission. After transfusion hemoglobin was 77 g/dL. A further 2 units of packed red blood cells was transfused 3 days late in response to a hemoglobin of 68. Hemoglobin level after the second transfusion was 89 g/dL. Upper GI endoscopy revealed varices and 6 bands were applied. There appeared to be jejunitis but gastric and jejunal biopsies were normal. Ophthalmology was consulted and examination for Kayser Fleischer rings was negative.

**Figure 1:**
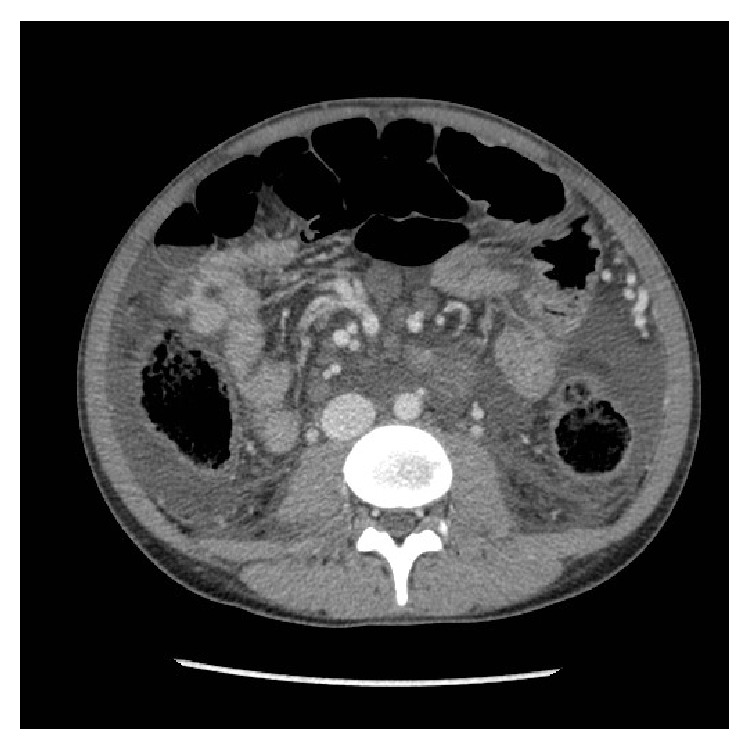
Axial view of abdomen showing ascites.

**Figure 2:**
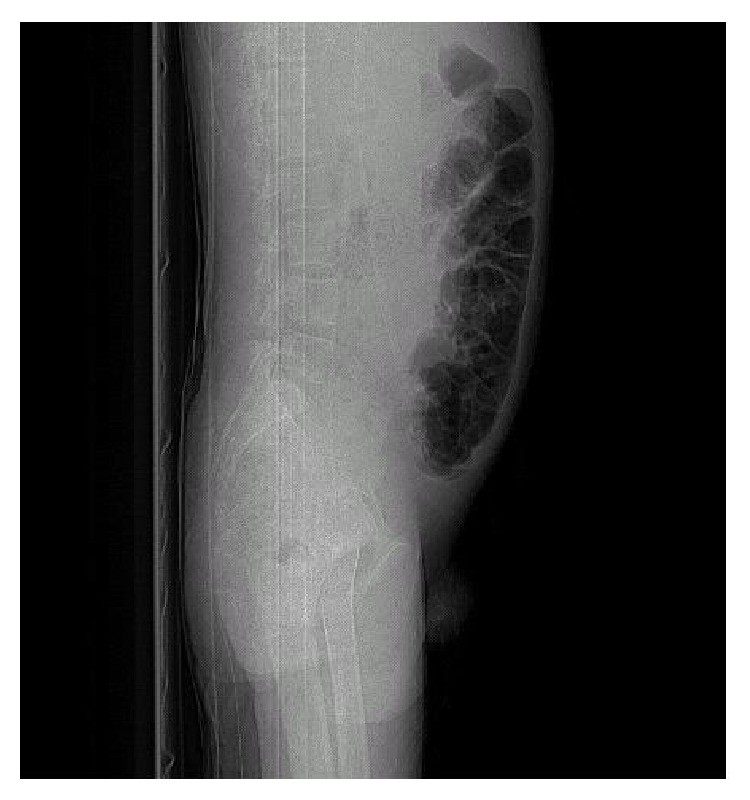
Lateral abdominal XR showing abdominal distension.

Cirrhosis was visualized on ultrasound and abdominal CT and a paracentesis was performed on day 2 of his admission. Peritoneal fluid cultures were negative for Acid Fast Bacilli, bacterial growth, or fungus. Scant neutrophils were reported. Marked distension secondary to ascites was present (Figures 1 and 2) on CT. Peritoneal fluid analysis revealed an albumin 3 g/L, glucose 7.2 mmol/L, LD 21 IU/L, pH 7.00, and protein 7 g/L, triglyceride levels <0.10, and amylase 0.74 IU/L. In the differential count, WBC were 0.1 × 10^9^/L and RBC were <0.01. Serum albumin levels on the day of the tap were 28 g/L. Invasive hepatic venous pressure gradients were not performed.

An ultrasound guided hepatic biopsy revealed cirrhosis and a diagnosis of Progressive Familial Intrahepatic Cholestasis (PFIC). The patient was discharged on spironolactone, nadolol, ursodeoxycholic acid, and pantoprazole. Serial surveillance endoscopies were planned. An appointment for follow-up and transplant work-up with Hepatology was made.

## 4. Discussion

Cirrhosis is the result of the replacement of necrotic hepatocytes by fibrosis and nodule formation [2]. Chronic liver injury leads to inflammation, necrosis, and ultimately fibrosis. Stellate cells are presinusoidal contractile cells that undergo activation and result in the initiation of fibrosis [2–4].

A summary of the common causes is included as follows.

*Causes of Hepatic Injury and Cirrhosis*

First are inflammatory diseases.

- Infectious: viral hepatidities, EBV, and CMV.
- Parasitic: schistosomiasis.
- Autoimmune hepatitis.

Second are toxins.

- Prescribed: idiosyncratic reactions and known hepatotoxic agents.
- Recreational: alcohol.
- Over the counter: acetaminophen.
- Herbal: Chinese herbals and amanita mushrooms.
- Environmental: carbon tetrachloride.

Third are genetic/hereditary diseases.

- Wilson's disease, *α*-1 antitrypsin deficiency, hereditary hemochromatosis, primary biliary cirrhosis (PBC), and primary sclerosing cholangitis (PSC).

Fourth are vascular diseases.

- Venoocclusive disease, for example, Budd-Chiari syndrome and portal vein thrombosis.

Fifth are infiltrative diseases.

- Sarcoidosis.
- Nonalcoholic steatohepatosis (NASH).

Sixth are idiopathic cases.

Cirrhosis affects approximately 5 million individuals in the United States at a cost of more than $4 billion annually. The incidence is estimated to be 360 cases per year per 100,000 of population. It is the 11th leading cause of death and accounts for more than 30,000 deaths per year. Cirrhosis is one of the leading causes of death in people over age of 65 years. The two most common causes of cirrhosis in the United States are alcoholic liver disease and Hepatitis C [5–7]. A thorough patient history is important to identify the possibility of any reversible etiology of the liver injury and cirrhosis.

Severe portal hypertension and cirrhosis can result in a spectrum of manifestations from asymptomatic to multisystem dysfunction. Symptoms of hepatic decompensation include jaundice, pruritus, hematemesis, melena, ascites, encephalopathy, easy bruising, and menstrual abnormalities [6]. Physical signs include jaundice, spider angiomata, gynecomastia, ascites, splenomegaly, palmar erythema, digital clubbing, asterixis, peripheral edema, and ascites [6].

Infectious etiologies can be ascertained with a combination of history and laboratory investigations. History of travel to Africa or South America is a risk factor for* Trypanosoma mansoni* infection and stool and urine specimens should be collected for parasite detection. A history of intravenous drug use, unsafe sexual practices, and blood transfusions are risk factors for Hepatitis B and Hepatitis C. It is challenging to diagnose EBV and CMV infections clinically. Travel to endemic areas of Hepatitis A or any history of jaundice is a strong indication to order serology of the viral hepatidities as well as EBV and CMV [2–4, 7, 8].

Autoimmune hepatitis typically follows a bimodal incidence with peaks in female patients in their teens or in the perimenopausal years. Concomitant autoimmune disorders (e.g., thyroiditis or connective tissue disorders) may be noted. There is also overlap with primary biliary cirrhosis and primary sclerosing cholangitis. Prevalence is 1 : 6000–1 : 7000. Three types of autoimmune hepatitis have been recognised. Type I autoimmune hepatitis has positive anti-nuclear antibodies (ANA) and positive anti-smooth muscle antibodies (antiactin). Type II findings include positive anti-liver kidney antibodies (anti-LKM1). Type III behaves like Type I and soluble liver antigen (anti-SLP/LP) is found [2–4, 8].

Review of potentially hepatotoxic drugs should be conducted but the medication history should also include nonprescribed medications. Hepatotoxicity to prescribed drugs may be idiosyncratic but numerous commonly prescribed drugs may contribute to the development of cirrhosis or precipitate deterioration. A complete discussion on hepatotoxic drugs is beyond the scope of this paper but antibiotics (e.g., tetracycline), antiretrovirals (e.g., AZT), anesthetic agents (e.g., halothane), antituberculosis medications (e.g., isoniazid), antiepileptics (e.g., valproate), and chemotherapeutic agents like methotrexate are all potentially hepatotoxic [2–4, 8]. Nonprescribed agents are often implicated in cirrhosis. Excessive alcohol consumption is one of the major causes of cirrhosis. It is important to obtain an acetaminophen history. Overdose situations are potentially treatable but delay in diagnosis can cause irreversible liver damage or death. A history of occupational exposure to carbon tetrachloride in refrigeration or dry-cleaning may be important to elucidate. Traditional medications like Chinese herbals and accidental ingestion of amanita mushrooms should be questioned [2–4, 8].

Numerous genetic conditions predispose patients to the development of cirrhosis. Wilson's disease has a homozygote incidence of 1 : 30 000–1 : 300 000. An abnormality in liver copper metabolism results in an inability for the liver unable to prepare copper for biliary excretion. There is no definitive test for Wilson's disease but the presence of Kayser Fleischer rings, low copper ceruloplasmin levels, high urine copper, and high copper content on liver biopsy are all highly suggestive of the condition [2–4, 8]. Alpha-1 antitrypsin deficiency is not rare, with an incidence of 1 : 1600–1 : 2800 but it does cause cirrhosis. It is commonly first diagnosed as a result of pulmonary involvement that manifests as bronchiectasis or emphysema; however, the intrahepatic accumulation of abnormal protein leads to liver disease. Serum levels of alpha-1 antitrypsin can vary and definitive diagnosis is by genetic testing [2–4, 8]. Hemochromatosis is an autosomal recessive condition that results in pathological deposition of iron in the liver, pancreas, and heart. Concomitant diabetes and cardiomyopathy may be present. Homozygote frequency is 1 : 200–1 : 400. High serum ferritin levels and transferrin saturation greater than 45% are highly suggestive of hemochromatosis. Mutation analysis of the HFE gene is helpful to confirm the diagnosis but utility may be limited if the hemochromatosis is not due to an HFE mutation. Liver biopsy remains a useful adjunct to confirming the diagnosis [2–4, 8].

A lower threshold for considering or suspecting primary biliary cirrhosis (PBC) should be held for female patients with concomitant autoimmune diseases. An elevated ALP, high IgM, low albumin, and prolonged PT are nonspecific indicators of the presence of the disease. Antimitochondrial antibodies are positive in 90–95% of patients [2–4, 8]. Primary sclerosing cholangitis (PSC) is associated with up to 70% of patients with inflammatory bowel disease. ALP is elevated and a positive ANCA is present in 60–70% of cases. ERCP or MRCP can confirm the changes in biliary anatomy [2–4, 8]. Cirrhosis may result from vascular problems such as venoocclusive disease. Budd-Chiari syndrome is caused by venous-outflow obstruction due to occlusion of the hepatic vein. This may be due to hypercoagulable states including polycythemia rubra vera, leukemia, or other more commonly heritable conditions. Mass effect from abdominal tumors or hydatid cysts may also disrupt the flow sufficiently to cause occlusion. Investigations that assist in diagnosis include a high protein content of ascitic fluid, and abnormal blood flow in the hepatic vein on abdominal ultrasound [2–4]. Infiltrative causes of cirrhosis include sarcoidosis and nonalcoholic steatohepatosis (NASH). The multisystem granulomatous manifestations of sarcoidosis more commonly include pulmonary, dermatological, or ocular involvement. Screening tests that may suggest the presence of sarcoidosis and support the pursuit of a tissue biopsy include elevated Angiotensin Converting Enzyme (ACE) levels, mild hypercalcemia, and hypergammaglobulinemia. The concomitant presence of obesity, hypercholesterolemia, and glucose intolerance and the absence of other conclusive laboratory findings suggest that NASH is responsible for the cirrhosis [2–4]. Up to 30% of cases of cirrhosis may be cryptogenic or idiopathic despite thorough work-up [7].

The portal vein is formed by the union of the superior mesenteric vein and splenic vein. The normal pressure is 5–8 mmHg and there is little gradient across the liver to the hepatic vein. Portal hypertension can be divided into prehepatic (e.g., portal vein thrombosis), intrahepatic (e.g., cirrhosis or hepatitis), and posthepatic causes (e.g., Budd-Chiari syndrome, right heart failure, or constrictive pericarditis).

The management of cirrhosis and portal hypertension is summarized in Treatment of Cirrhosis and Portal Hypertension. General measures include presenting and mitigating further damage and treating the underlying cause. More specific therapies are tailored to treating specific complications.

*Treatment of Cirrhosis and Portal Hypertension*

*General Measures*

Treat underlying cause.

Remove hepatotoxic medications/agents.

*Specific Complications*

Ascites:

 sodium restriction <60–90 meq/day (1.5–2 g of salt/day) and fluid restriction (<1000 mL/day),
 medical measures: low dose diuretics (spironolactone 50–200 mg/day and amiloride 5–10 mg/day),
 low dose furosemide (caution is a risk of excessive diuresis): target weight loss is 300–500 g/day in patients without peripheral edema and 800–1000 g/day in patients with peripheral edema.

Paracentesis:

 transjugular intrahepatic portosystemic shunt (TIPS).

Spontaneous bacterial peritonitis (SBP):

 cephalosporins (acute therapy),
 quinolones (prophylaxis).

Hepatorenal syndrome:

 removal of nephrotoxic agents and correction of hypovolemia,
 vasopressin analogues and alpha-adrenergic agents (e.g., midodrine) in combination with albumin.

Hepatic encephalopathy:

 lactulose,
 rifaximin.

Variceal hemorrhage:

 emergent management: acute resuscitation, blood transfusion, IV antibiotics, IV octreotide, and esophagoduodenoscopy,
 longer term management: nadolol and TIPS.

See [4, 9–11].

Progressive Familial Intrahepatic Cholestasis (PFIC) refers to a group of autosomal recessive disorders that disrupt bile formation. Three types of PFIC have been identified. PFIC1 and PFIC2 typically present in childhood. PFIC3 may manifest later in infancy or childhood or, as in this case, in adulthood. Defects in the ABCB11 gene, encoding multidrug resistance 3 (MDR3) protein, impair biliary phospholipid secretion. Moderate pruritus may be reported. Biochemical findings include elevated GGT, mildly elevated ALT, normal ALP, and high serum bile acid concentration. Liver histology reveals ductular proliferation and biliary fibrosis [12–15]. PFIC3 is functionally a cholestatic pathology.

Ursodeoxycholic (UDCA) acid is a nontoxic hydrophilic bile acid that protects hepatocytes and cholangiocytes by replacing endogenous cytotoxic bile salts. It is also proposed that UDCA increases hepatocyte excretion of bile acids and limits return to the liver by inhibiting their intestinal reabsorption. It is one of the very few specific medical options available to manage PFIC. Up to 46% of patients with PFIC3 respond to UDCA [14, 15].

General symptomatic management of the cholestatic patient includes management of pruritus. The mechanism poorly is understood but may be due to the accumulation of hydrophobic bile acids or endogenous opioids. Intractable pruritus can lead to liver transplantation. In the absence of bile duct obstruction amenable to treatment, management of pruritus typically involves oral therapies due to the poor efficacy with topical treatments [14, 15]. Intractable symptoms may lead to transplantation. Cholestyramine, an anion-exchange resin, is first line management and up to 80% cases of cholestatic patients respond. Rifampin is effective in approximately 50% of patients. Naltrexone, nalmefene, and sertraline can also be considered [13–15]. Fatigue is a common symptom in cholestatic disease. The pathogenesis of fatigue in cholestasis is poorly understood but possibly involves changes in central neurotransmission, which result from signalling between the diseased liver and the brain. There is a poor correlation with degree of fatigue and stage of cholestatic disease [13]. A management approach includes ruling out potentially contributing factors like depression, anemia, thyroid dysfunction, renal dysfunction, and sleep disturbances. Management options are limited and no therapies have been identified to be clearly beneficial. Emphasis should be placed on stress reduction, healthy lifestyle, avoidance of alcohol and caffeine, regular exercise, and adequate sleep [13, 14].

Osteoporosis occurs in cirrhosis of all etiologies. Increased resorption and decreased formation of bone contribute to the development of osteoporosis in patients with cholestasis. Calcium and vitamin D supplementation should be encouraged and bisphosphonate therapy has proven benefit in patients with PBC [13, 14].

Surgical management of PFIC includes biliary diversion. External diversion entails linking gallbladder drainage to skin via a stoma and internal drainage involves gallbladder drainage to colon. The net result is bypassing of the terminal ileum where bile salts are reabsorbed. Liver transplant is the last option if severe cirrhosis is present and the patient has failed to improve with UDCA and biliary diversion [13, 14].

## 5. Conclusion

Cirrhosis is a significant cause of morbidity and mortality. Although up to 30% of cases may be diagnosed as idiopathic, there are numerous reversible etiologies that should be identified and treated. The clinical course may vary from asymptomatic to life-threatening. Available therapies mainly help with symptom control. Early and accurate diagnosis can delay clinical decline and possibly the need for transplantation.

## Figures and Tables

**Table 1:** Summary of infectious work-up.

Monospot test	Negative
Hepatitis A IgG	Positive
Hepatitis C	Negative
Hepatitis B IgG	Positive
Hepatitis B sAG	Negative

Epstein-Barr virus VCA IgG	Positive
Epstein-Barr virus VCA IgM	Negative
Epstein-Barr virus NA IgG	Positive

Parvovirus B19 IgG	Negative
Parvovirus B19 IgM	Negative

Cytomegalovirus IgG	Negative
Cytomegalovirus IgM	Negative

RPR	Negative
